# Within-hour incremental movement data of finishing pigs over 20 days using RFID–LoRaWAN technology

**DOI:** 10.1038/s41597-026-07469-9

**Published:** 2026-05-25

**Authors:** Marko Ocepek

**Affiliations:** Norwegian University of Life Sciences, Faculty of Biosciences, Department of Animal and Aquacultural Sciences, PO Box 5003, 1432 As, Norway

**Keywords:** Behavioural methods, Animal behaviour

## Abstract

This Data Descriptor presents an openly available movement dataset collected from 16 finishing pigs during the final 20 days before slaughter using a custom RFID and LoRaWAN monitoring system. The released CSV file (MOVEMENT_Final.csv) contains 1,048,573 repeated within-hour movement increment records rather than one pre-calculated hourly total per pig and hour. Each record includes pig identifier, study day, hourly block start time, and incremental movement in cm. In complete pig-day-hour blocks, the file typically contains about 180 records, consistent with an approximately 20-second processed output interval. Hourly totals can be reconstructed by summing distance values within each pig × day × hour block, yielding 6,026 valid hourly observations out of 7,680 theoretically possible blocks. The dataset supports research on activity rhythms, behavioural variability, preprocessing workflows for livestock sensor data, and precision livestock farming. Complete data gaps occurred at the study boundaries because of technical interruptions and should be treated as missing rather than zero activity.

## Background & Summary

Quantifying animal movement is important for studies of behaviour, welfare, and precision livestock farming because temporal changes in activity may reflect feeding, resting, exploration, disturbance, or emerging health problems. Continuous sensor-based monitoring can provide a richer temporal picture than occasional manual observations, especially under commercial conditions^1–3^.

In this study, pigs were monitored with a custom-built RFID and LoRaWAN system. RFID provides animal-level identification when a tagged animal is detected in the reading field of a receiver, whereas LoRaWAN enables low-power, long-range wireless transmission of those records to a gateway and database. Such architectures are increasingly attractive for livestock applications because they support automated and relatively low-maintenance data collection^4,5^.

The dataset was collected on a commercial finishing pig farm in Norway from 2 February to 21 February 2023, corresponding to the last 20 days before slaughter. Sixteen pigs were individually monitored within one deep-straw pen. The present Data Descriptor documents the released dataset itself, its structure, limitations, and how hourly summaries are derived from the repeated within-hour records^6^.

A key clarification for reuse is that the public CSV file is not a one-row-per-hour dataset. Instead, it contains repeated movement records nested within hourly blocks. This structure explains why the file contains more than one million rows even though the theoretical number of pig × day × hour combinations is much smaller. Because this distinction is essential for correct reuse, this Data Descriptor explicitly documents the released file structure and the reconstruction of hourly totals^6^.

## Methods

### Animals and housing

The study was conducted in a Norwegian commercial deep-straw finishing unit during the final 20 days before slaughter. The barn contained three identical pens, each housing 32 finishing pigs. Data collection was performed in one pen measuring 14.3 m × 4.8 m (68.6 m²), corresponding to approximately 2.14 m² per pig at the pen level. Within that pen, 16 of the 32 pigs were equipped with passive RFID ear tags and individually monitored throughout the study period. The monitored animals were of mixed sex (8 barrows and 8 gilts). The pen had a solid concrete floor covered with deep straw bedding, replenished weekly with one straw bale, a four-space dry feeder, four drinking bowls, mechanical ventilation, and ad libitum access to feed and water.

### RFID and LoRaWAN system

Pig movement was monitored using a custom RFID-based sensor network installed in the barn. The monitored pen was equipped with a central receiver cabinet mounted at the midpoint of the long wall. The cabinet contained the protected power supply, receiver hardware, and a communication unit with three antennas arranged as one central antenna and one antenna on each side, enabling wireless data transmission and transfer. Fixed receivers and external antenna components were connected to the cabinet, and detections were logged as time-stamped records with receiver identity and received signal strength indicator (RSSI). Based on the system layout and on-site testing, the practical coverage extended approximately 7.15 m in each direction along the pen’s long axis, corresponding to coverage of the full 14.3 m pen length, although detections were most reliable in the wall-side zone closest to the receiver setup. The monitored functional areas therefore included the feeder side, drinker side, and the central resting/exploration area within the covered pen.

### Data acquisition and processing

Time-stamped detections were first collected as event-based records. In the analytical workflow used for data processing, detections were converted to distance-to-receiver estimates using an empirical log-distance model based on RSSI:

$${distance}={10}^{(({TxPower}-{RSSI})/(10\times n))}$$

where T*x*Power is the assumed received signal strength at 1 m and n is the path-loss exponent estimated from calibration measurements. Consecutive distance estimates for each pig were then used to derive incremental movement, defined as the absolute change between successive distance estimates. Hourly movement was obtained by summing these incremental values within each pig × day × hour block.

The released file MOVEMENT_Final.csv contains repeated within-hour records rather than a single final hourly total per block. The file has four columns: pid_id, Day_s, distance, and Hour. The Hour column represents the start time of the hourly block in HH:MM:SS format. In complete blocks, the public file usually contains 180 records and occasionally 181 records, consistent with an approximately 20-second processed output interval in the released dataset. By summing the distance values within each pig × day × hour block, the public file can be aggregated into 6,026 valid hourly observations, matching the derived hourly dataset used in downstream analyses.

Hours without valid positioning data were excluded from inferential analyses. These missing hours indicate insufficient detections for deriving movement and should not be interpreted as confirmed inactivity.

## Data Records

The dataset is openly available at Zenodo^6^ as MOVEMENT_Final.csv and is distributed under a Creative Commons Attribution 4.0 International licence. The released CSV file contains repeated within-hour movement increment records rather than pre-aggregated hourly totals (Table 1).

**Table 1:** Description of variables in MOVEMENT_Final.csv.

Variable	Description
pid_id	Unique pig identifier (string)
Day_s	Consecutive day number within the 20-day observation period(1–20, integer)
Hour	Start time of the hourly block in HH:MM:SS format (e.g. 13:00:00 = beginning of 13:00–13:59 block)
distance	Incremental movement (cm) within the ~20-second processed output interval – to obtain the total hourly movement (cm/h), sum all distance values within the same pid_id + Day_s + integer hour (extract hour from Hour timestamp)

In the public file there are 1,048,573 rows in total, which collapse to 6,026 unique pig × day × hour blocks after aggregation by summing the distance column. This distinction is critical for correct reuse: the released CSV contains multiple repeated rows within the same hour (typically ~180 in complete blocks, approximately evenly spaced at 20-second intervals during active periods), whereas the derived hourly table contains one aggregated value per pig × day × hour block. The dataset does not include a separate column for exact sub-second timestamps; the Hour column marks the block start time, and records are generated at roughly regular intervals in periods with detections.

## Data Overview

After aggregation of the released CSV by pig × day × hour, the dataset contains 6,026 valid hourly observations out of 7,680 theoretically possible blocks. The mean hourly movement was 792.8 cm/h, the minimum was 0.0 cm/h, and the maximum was 1439.9 cm/h. The maximum occurred for pig ET00008 on day 8 during the 13:00 hour block. Low or zero values in valid hourly blocks indicate low estimated locomotion within covered areas, whereas missing hourly blocks indicate insufficient valid detections.

Dataset completeness differed across pigs. Twelve pigs contributed 449 to 451 valid hourly observations, one pig contributed 321 hours, two pigs contributed 139 and 152 hours, and one pig contributed only 4 valid hourly observations. These completeness differences should be considered when modelling between-pig differences.

There were also complete day-hour gaps across all monitored pigs. No pig-level records were available for study day 1 from 12:00 to 23:00 and for study day 20 from 00:00 to 16:00. These periods should be treated as technical interruptions rather than biological absence.

**Table:** 

Pig ID	Valid hourly observations
ET00058	4
ET00016	139
ET00018	152
ET00008	321
ET00088	449
ET00010	451
ET00037	451
ET00033	451
ET00038	451
ET00066	451
ET00085	451
ET00086	451
ET00089	451
ET00095	451
ET00096	451
ET00098	451

## Technical Validation

Calibration and validation were performed before the main recording period using a pilot dataset generated by moving tagged transponders across known positions and distances within the monitored pen. RSSI values were converted to estimated distance using an empirical log-distance model:

$${\rm{distance}}={10}^{(({\rm{T}}{\rm{x}}{\rm{Power}}-{\rm{RSSI}})/(10\times {\rm{n}}))}$$

where TxPower is the assumed received signal strength at 1 m and n is the path-loss exponent. Both parameters were derived from the calibration measurements and then applied to the main dataset. Because RSSI-based distance estimates are sensitive to tag orientation and multipath effects, particularly at longer ranges, the pilot calibration and on-site system testing were used to confirm that changes in estimated distance corresponded to known movements under barn conditions.

During the recording period, the system was checked daily at the operational level to confirm continued function of the cabinet, power supply, receiver hardware, wireless communication, and incoming ear-tag detections. During preprocessing, duplicated or invalid detections and hours without sufficient valid positioning information were identified and excluded from the hourly analytical dataset. Cross-checking the public CSV against the derived hourly analytical table confirmed an exact one-to-one match after summation within each pig × day × hour block.

Because RSSI-based distance estimation is sensitive to tag orientation, multipath effects, and coverage limits, users should interpret the data as a movement proxy within the covered zone rather than as an exact two-dimensional trajectory. Hours without valid positioning data were excluded rather than imputed. This avoids overstating certainty during technical interruptions but also means that data completeness differs across pigs and time. Quantitative validation metrics from the pilot phase were not retained in the archived technical records; therefore, the present description reports the calibration workflow, practical coverage, and preprocessing procedure rather than unsupported precision estimates.

To facilitate reproducibility, the Zenodo deposition6 was updated to version 1.1 with a basic Python aggregation code block, allowing users to sum incremental distances to hourly totals without custom preprocessing. In addition to processing validation, internal consistency checks were performed on the released dataset. The distribution of within-hour movement increments was right-skewed, with many small increments and fewer larger values, consistent with the expected structure of movement data.

After aggregation within pig × day × hour blocks, the resulting hourly movement values reproduced the derived analytical dataset exactly and fell within plausible observed ranges. Missing-data patterns were also consistent with known technical interruptions at the study boundaries rather than isolated random gaps across all pigs.

## Usage Notes

The public CSV is best used in one of two ways. First, it can be analysed directly as a within-hour movement-record dataset for methodological work on aggregation, filtering, or sensor-data preprocessing. Second, it can be aggregated to one hourly value per pig × day × hour block by summing the distance column, thereby reproducing the derived hourly movement table.

Users should not assume that one row in MOVEMENT_Final.csv equals one hourly total. They should also account for known completeness differences among pigs and for the complete technical gaps on day 1 from 12:00 onward and on day 20 before 17:00. To facilitate reproducibility, a basic Python aggregation code block is included in the Zenodo description^6^, allowing users to sum incremental distances to hourly totals without custom preprocessing. The dataset is intended primarily for methodological development and exploratory analysis rather than strong biological inference.

## Data Availability

The movement dataset is openly available at Zenodo^6^ (updated to version 1.1 in March 2026 to align the title, metadata, and description with the repeated within-hour incremental structure and include aggregation instructions and code block). The released file, MOVEMENT_Final.csv, contains repeated within-hour movement increment records grouped by pig, day, and hour block.
